# Relevance of Brain ^18^F-FDG PET Imaging in Probable Seronegative Encephalitis With Catatonia: A Case Report

**DOI:** 10.3389/fpsyt.2021.685711

**Published:** 2021-06-09

**Authors:** Michaël Guetta, Aurélie Kas, Aveline Aouidad, Marine Soret, Yves Allenbach, Manon Bordonné, Alice Oppetit, Marie Raffin, Dimitri Psimaras, David Cohen, Angèle Consoli

**Affiliations:** ^1^Department of Child and Adolescent Psychiatry, APHP. SU, Pitié-Salpêtrière Hospital, Paris, France; ^2^Department of Nuclear Medicine, APHP. SU, Pitié-Salpêtrière Hospital, LIB, INSERM U1146, Paris, France; ^3^Sorbonne-University, Internal Medecine and Clinical Immunlogy Departement, Paris, France; ^4^University of Lorraine, Department of Nuclear Medicine and Nancyclotep Imaging Platform, CHRU Nancy, Nancy, France; ^5^Department of Neurology, APHP. SU, Pitié-Salpêtrière Hospital, Paris, France; ^6^CNRS UMR 7222, Institute for Intelligent Systems and Robotics, Sorbonne University, Paris, France; ^7^National Center for Rare Psychiatric Diseases, APHP. SU, Pitié-Salpêtrière Hospital, Paris, France

**Keywords:** adolescence, catatonia, cerebral PET/CT, encephalitis, abnormal movement

## Abstract

Autoimmune encephalitis (AIE) is a rare, severe, and rapidly progressive encephalopathy, and its diagnosis is challenging, especially in adolescent populations when the presentation is mainly psychiatric. Currently, cerebral 18-fluorodeoxyglucose positron emission tomography (^18^F-FDG-PET) imaging is not included in the diagnosis algorithm. We describe a 16-year-old patient with probable seronegative encephalitis with catatonia for which several cerebral PET scans were relevant and helpful for diagnosis, treatment decision making, and follow-up monitoring. The patient recovered after 2 years of treatment with etiologic treatment of AIE and treatment of catatonia. This case suggests a more systematic assessment of the clinical relevance of ^18^F-FDG-PET imaging in probable seronegative AIE.

## Introduction

Autoimmune conditions associated with psychosis and catatonia include systemic autoimmune disorders that can target the central nervous system (e.g., systemic lupus erythematosus) and autoimmune encephalitis (AIE) (1, 2). Autoimmune encephalitis (AIE) is a rare, severe, and rapidly progressive encephalopathy caused by antibody-mediated neuroinflammation (3). AIE is a form of encephalitis, which are diseases involving severe brain inflammation with different causes and a complex differential diagnosis (3). Diagnosing AIE is challenging because of the overlap in clinical presentations between AIE, other inflammatory brain diseases, brain infections, metabolic diseases, and psychiatric disorders (1). It is especially difficult in children because of the complexity of normal behavioral changes during childhood and the limited capacity of younger children to describe their symptoms (4). Moreover, compared to adults with AIE, children may manifest important differences in symptoms, paraclinical findings, comorbidities, treatment response, and prognosis (5, 6). They may present with a wide array of psychiatric symptoms, including psychosis, obsessive compulsive disorder, mania, hypomania, catatonia, autistic/cognitive regression, and other conditions (7).

Several antibodies have been described in children with AIE (5), and the three most commonly noted antibodies target the N-methyl-D-aspartate receptor (NMDAR), myelin oligodendrocyte glycoprotein (MOG), and glutamic acid decarboxylase 65 (GAD65) (6, 8–10). It has also been recognized that not all children with a clinical phenotype of AIE have a known autoantibody (4). A consensus statement paper proposed defining three diagnostic categories of “possible,” “probable,” and “definitive” AIE. The paper proposed specific diagnostic criteria mixing clinical and paraclinical signs (3). However, to date, cerebral 18-fluorodeoxyglucose positron emission tomography (^18^F-FDG-PET) is not included in the diagnostic algorithm of AIE, even though few articles over the past 10 years have suggested that ^18^F-FDG-PET may contribute to early diagnosis (11–13) or contribute when no antibodies targeting the central nervous system are found (14, 15). Here, we present a case report of a 16-year-old boy with probable seronegative AIE in which several ^18^F-FDG-PET images were relevant and helpful for diagnosis, treatment decision making, and follow-up monitoring.

## Case Description

Jason is a 16-year-old boy with no medical or psychiatric history except repeated ear infections as a child. The first symptoms appeared at the age of 14 with a slight psychomotor slowdown and a lack of motivation during the summer. Six months later, the psychomotor slowdown worsened, and several neuropsychiatric symptoms appeared: abnormal movements of the upper limbs, fingers, and head and obsessive compulsive disorder with the impossibility of swallowing his saliva (cf. Supplementary Video 1). Within a few months, the patient developed a catatonic syndrome with catalepsy, posturing, progressive mutism, negativism, and a refusal to eat, which caused a loss in body weight of 10 kg.

Jason was first hospitalized in a neuropediatric department where he underwent exhaustive biological evaluations, brain MRI, several EEG recordings, video EEG, and lumbar puncture, which included a search for brain-targeted antibodies. All exams were normal, with the exception of moderate elevations in anti-streptolysin O (ASLO) and streptodornase antibodies. In this department, he was treated with a combination of benzodiazepine (lorazepam 12 mg/d) and amoxicillin (3 g/d for 3 weeks) due to a suspicion of PANDAS (Pediatric Auto-immune Neuropsychiatric Disorders Associated with Streptoccocus Infections), and this treatment led to a slight and very short improvement.

**Figure 1:**
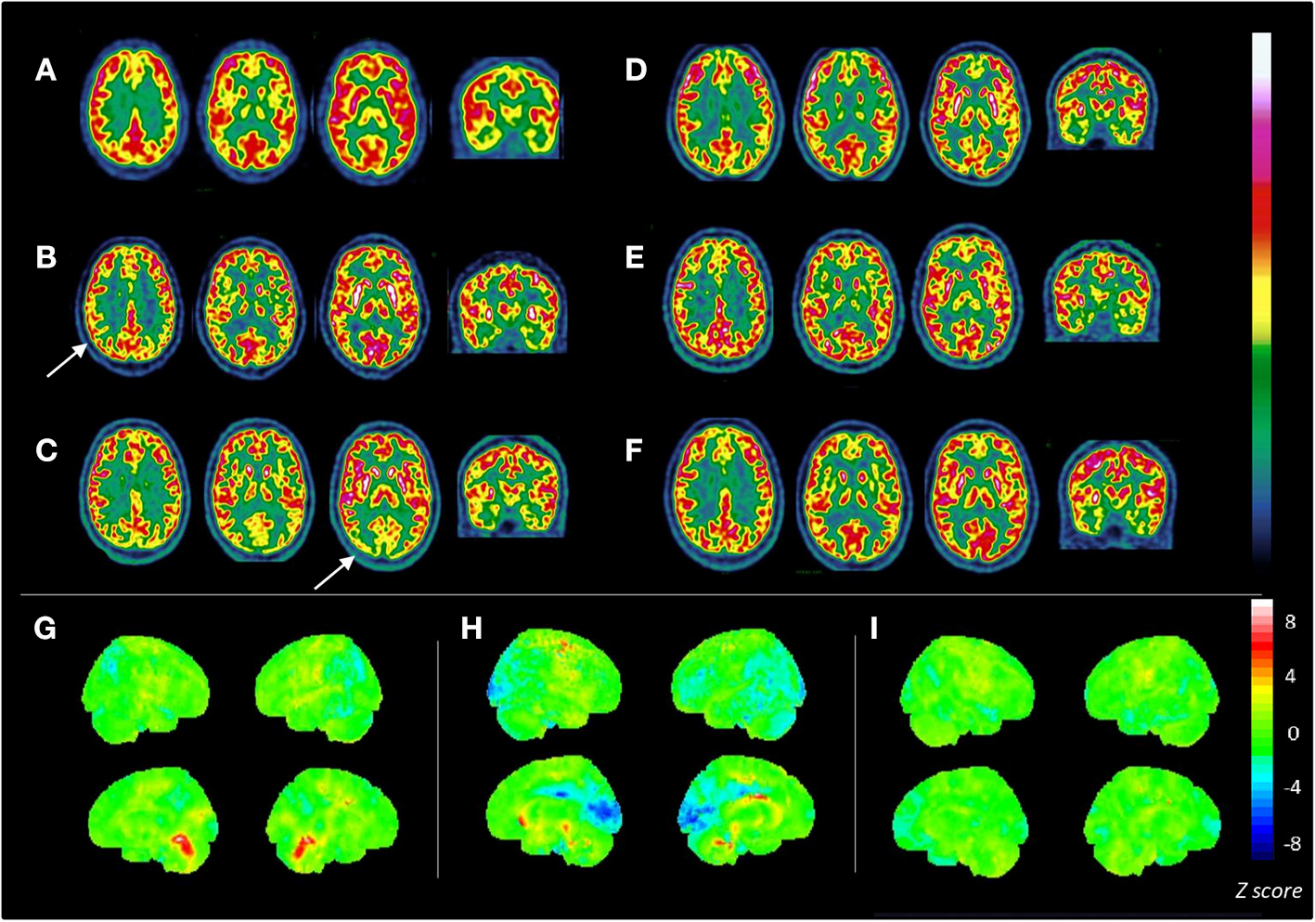
Consecutive brain ^18^F-FDG PET scans in a patient aged 16 y/o with seronegative AIE. Axial slices of 6 consecutive ^18^F-FDG PET scans **(A–F)** are displayed in radiological view with the French scale color. **(A)** First ^18^F-FDG PET: moderate mediofrontal and thalamic hypometabolism; ^18^F-FDG PET was performed after the first plasma exchange, when the patient's state was slowly improving. **(B)** Second ^18^F-FDG PET: moderate, bilateral parietotemporal hypometabolism (white arrow); bilateral striatal hypermetabolism; metabolism is significantly decreased compared with healthy controls in the posterior cingulate and parietotemporal cortices with Z-scores ranging from −2.1 to −2.9, and highly increased in the striatum (*Z*-score: +3.5) and cerebellar vermis (*Z*-score: +3.3) **(G)**; ^18^F-FDG PET was performed after the second plasma exchange that was ineffective. **(C)** Third ^18^F-FDG PET: deeper and widespread metabolism decreases in posterior cortex spreading to the primary visual cortex with *Z*-scores ranging from to −2.1 to −5.1 [in blue **(H)**]; striatal hypermetabolism [in red **(H)**]; deterioration of patient state; ^18^F-FDG PET was performed after the third plasma exchange that was inefficient. **(D)** Fourth ^18^F-FDG PET: partial improvement in posterior (*Z*-score: −2.3) and primary visual area metabolism (*Z*-score of −2.1 vs. a previous score of −5.1); occurrence of moderate mediofrontal and hippocampal hypometabolism; ^18^F-FDG PET was performed when the patient improved after rituximab, levodopa, ECT, and benzodiazepine administration. **(E)** Fifth ^18^F-FDG PET: normalization of bilateral posterior associative metabolism; normalization of striatal metabolism; stability of mediofrontal and mesiotemporal hypometabolism; ^18^F-FDG PET was performed when the patient was much improved, showing minor frontal syndrome and little abnormal movement. **(F)** Last ^18^F-FDG PET: examination showing mild, persistent mediofrontal and mesiotemporal hypometabolism; normal metabolism in the posterior cortex [as shown on the statistical map **(I)**]. **(G–I)** Show 3D statistical maps of voxel-based analyses for 3 ^18^F-FDG PET scans (**B,C,F**, respectively) in comparison with a control group <40 y/o (*n* = 23, mean age 30.9 ± 8.6 y/o; Scenium software, Siemens healthcare). Rainbow color scale indicates *Z*-score values. Medial and lateral 3D views of the right (R) and left (L) hemispheres are shown.

After this first-line treatment, he was transferred to our child and adolescent psychiatry department. A multidisciplinary team meeting including psychiatrists, neurologists, and internists led to the diagnosis of probable seronegative encephalitis. He was treated with six plasma exchanges (PE), an intravenous immunoglobulin (IVIG) cure, and five IV boluses of methylprednisolone. In a few weeks, this treatment resulted in a dramatic improvement in his mental and general condition, with the recovery of eating and talking. One year after the first symptoms had appeared, he was able to go back home and school. Only mild OCD symptoms (handwashing) remained. Shortly after this second-line treatment, he underwent an initial brain ^18^F-FDG-PET examination (2 MBq/kg, 10-min acquisition started 30 min post injection) which was the first abnormal paraclinical exam showing moderate mediofrontal and thalamic hypometabolism (Figure 1A).

One month later, his clinical state worsened, and the catatonic syndrome with abnormal movement reappeared, leading to a second hospitalization. A third-line treatment with an IVIG cure followed by 5 PE briefly improved the symptoms. After a 2-month relapse, rituximab (600 mg × 2), an immunosuppressant treatment, was administered along with a third cure of 10 PE that did not improve the clinical condition of the patient. A second brain ^18^F-FDG-PET examination was performed and showed the appearance of a metabolic decrease in the parietotemporal associative cortex and a high metabolic increase in the striatum (Figure 1B). In addition, the whole-body imaging that was performed to screen for occult malignancy was negative.

**Figure 2:**
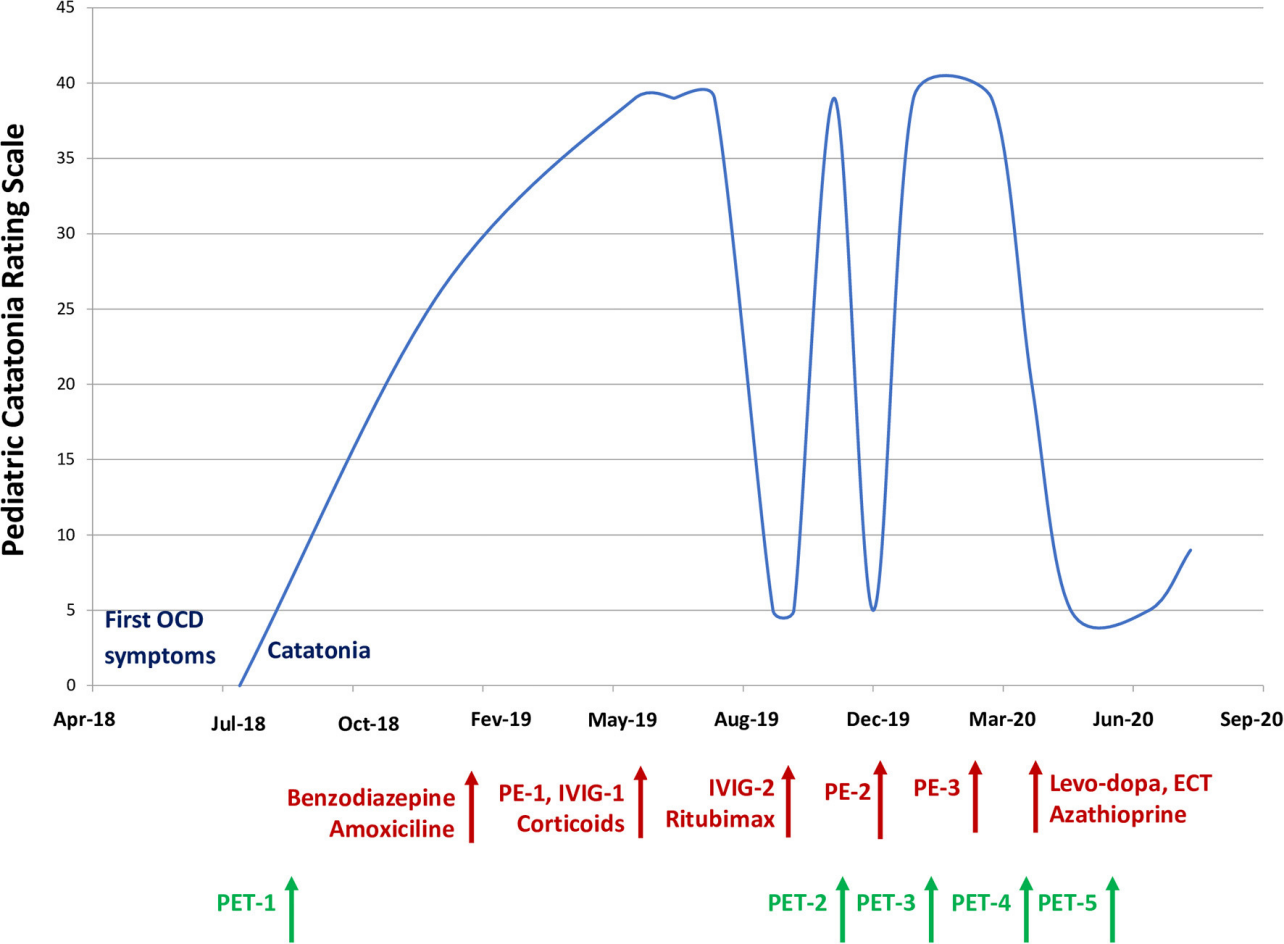
Pediatric catatonia rating scale based on time and treatment during the 2-year follow-up. OCD, obsessive compulsive disorder; PE, plasma exchanges; ^18^F-FDG PET, 18-fluorodeoxyglucose positron emission tomography; IVIG, intravenous immunoglobin; ECT, electroconvulsive therapy.

On brain ^18^F-FDG-PET performed 1 month later, the hypometabolism decrease worsened in the posterior areas and extended to the occipital cortex, including the primary visual cortex (Figure 1C). Once again, the ^18^F-FDG-PET findings were correlated with the patient's condition (Figure 2). During this hospitalization, a gastrostomy was performed because of refusal to eat and denutrition in the context of a very severe catatonic syndrome.

A few months later, a slow but important improvement of his clinical state appeared with the *effective* treatment of catatonia by lorazepam (20 mg) and ECT (6 sessions). It is likely that the late response to rituximab contributed to this improvement. A few weeks later, a second-line immunosuppressant (azathioprine) was introduced. He also received levodopa (187.5 mg/d) based on the hypothesis of impairments in the cerebral motivated behavioral system (basal ganglia, orbital cortex). Another ^18^F-FDG-PET performed to monitor the treatment response revealed a partial improvement in posterior associative metabolism, with the emergence of moderate mesiotemporal and mediofrontal hypometabolism (Figure 1D).

After 3 months, the improvement led to the recovery of eating, talking, catatonia (Figure 2) and Jason showed normal general behavior, although a slight frontal syndrome (lack of initiative and emotion, perseveration) and mild anosognosia persisted (cf. Supplementary Video 2). In agreement with the clinical course, brain metabolism returned to normal in the posterior areas and striatum with mild, residual hypometabolism in the mediofrontal cortex and hippocampus (cf. Figure 1E). Two months later, in August, a final cerebral ^18^F-FDG-PET scan was unchanged, with normal posterior associative cortex and mild medioprefrontal and mesiotemporal hypometabolism (Figure 1F).

During the writing of this case report, we had a follow-up with Jason and his family. He had a new relapse at the end of September with the recurrence of severe catatonic syndrome. No treatment improved his condition with the exception of the administration of the immunosuppressant rituximab that was promptly decided upon given Jason's history. In a few weeks, Jason was able to go back to school, although he sometimes had a brief psychomotor slowdown.

## Discussion

The diagnosis of AIE in children and adolescents is challenging for several reasons: (a) numerous differential diagnoses; (b) low incidence; and (c) changes in mood, behavior, and personality, possibly coinciding with puberty (3). Over one-third of patients with AIE present abnormal movements, such as ataxia, chorea, dystonia, myoclonus, or tremor (5, 16–18), and over 50% of patients present multifocal neuropsychiatric symptoms rather than isolated clinical syndromes (19). However, many present with psychiatric symptoms at an early stage.

In Jason's case, the first point that caught our attention was the diagnostic challenge. If we refer to the Graus et al. (3) consensus statement that proposed three diagnostic categories of “possible,” “probable,” and “definitive” AIE, the diagnosis of possible AIE was made in the beginning. Indeed, for this patient, we only had a limited number of atypical neurological symptoms associated with a psychiatric presentation: abnormal movements that appeared at the beginning of the illness were not specific to any neurological or psychiatric disease (cf. Supplementary Video 3). Because AIE can resemble acute psychosis and schizophrenia, children, and adolescents are sometimes referred to psychiatric services, causing concern about misdiagnosis of young patients with acute psychosis and not diagnosing underlying organic conditions (1, 20). In Jason's case, during multidisciplinary meetings to discuss the case, several psychiatric diagnoses were considered, such as melancholia and conversion disorder, which resulted in back and forth between the neurology department and psychiatric department, impacting the time until different treatments were administered. Currently, we know that the early diagnosis of pediatric AIE is crucial because delays in treatment worsen the prognosis and increase the risk of permanent neurocognitive deficits (21, 22).

To date, ^18^F-FDG-PET imaging is not included in the Graus et al. (3) criteria, although its contribution to limbic encephalitis is mentioned by the authors as a footnote in panel 2. However, we used the causality algorithm described by Ferrafiat et al. in 2018 to guide diagnosis and treatment for our patient. The CAUS score was 7/10, which was an argument for an autoimmune condition (threshold > 5) (15). In addition, if we consider Jason's brain metabolism abnormalities that correlated with clinical severity as relevant, then the diagnosis of probable AIE would be made. This is the hypothesis we had that justified aggressive immunomodulatory and immunosuppressive treatment resulting in significant clinical improvement (Figure 2). In a relevant number of patients described in the literature, ^18^F-FDG-PET imaging has shown limbic and extralimbic abnormalities, even in MRI-negative or inconclusive cases (12). Probasco et al. (13) demonstrated that in 61 adults with AIE, brain ^18^F-FDG-PET was more often abnormal (85% of the cases) than EEG, MRI, or routine CSF analysis. Brain hypometabolism was the most observed pattern. Solnes et al. demonstrated that brain ^18^F-FDG-PET had a better sensitivity than MRI in 23 (21 adults and 2 youths) seropositive patients with AIE. In these papers, the imaging pattern was more often a decrease than an increase in regional metabolism (12). These PET findings were also more strongly associated with the clinical picture and active disease status than MRI. The literature regarding PET and AIE is very limited in the pediatric population and is limited to case reports [e.g., (14, 15)]. The largest report is a retrospective study of 34 children showing that quantitative PET was abnormal in 94.1% of patients with acute AIE vs. 41.2% for MRI and 6.9% for CT (23).

We believe that the current case adds to this literature that suggests PET as a relevant exam for possible AIE. Throughout the period of support for this patient in our department, several ^18^F-FDG-PET scans were performed and helped us for several reasons: (a) the abnormalities in ^18^F-FDG-PET were an argument for an ongoing neurological impairment, even if it was not included in the diagnostic algorithm; (b) the improvement in brain metabolism matched patient improvement, and this correlation led us to be very determined in the use of aggressive immunomodulatory and immunosuppressive treatment. All consecutive ^18^F-FDG-PET examinations were performed in a single center with high experience in neuroimaging, and the images were interpreted in a double-blind manner by a second colleague to check interrater validity. In addition, as shown in Figure 1, we used a control group of 23 individuals aged <40 years with no neurologic or psychiatric symptoms to compare Jason's PET scans with the *Z*-scored method provided by Scenium software (Siemens Healthineers, Erlangen, Germany). Significant differences in metabolism were found in the brain regions interpreted as abnormal by nuclear medicine practitioners (see Figures 1G–I).

In the past few years, with the introduction of hybrid PET/MR systems, many groups have started to replace MRI with PET–MRI in diagnostic algorithms for other brain disorders, and we hypothesize that PET can become more commonly used for AIE diagnosis in the future (24). However, insurance barriers can also influence access to PET. Access is dependent on the institution, and it has not yet been included in any proposed diagnostic criteria or assessment algorithms, making efforts to obtain insurance prior authorizations particularly challenging in some countries.

We are aware of the limitation of a single case report regarding generalization of the findings. Additionally, the controls we used as comparisons for Jason's PET scans using the *Z*-scored method were not adolescents with typical development but young adults. Indeed, such a database is not available, as it would require the injection of radioactive compounds in healthy children or adolescents, which is not ethically possible. In addition, we cannot clarify the role of each treatment in the great improvements in the patient's clinical condition because he received both etiologic (e.g., PE) and symptomatic (e.g., ECT) treatments at the same time. Additionally, treatment response timing may dramatically differ among the treatments. As an example, there is a quasi-immediate response regarding catatonia under high-dose benzodiazepine treatment (25), whereas the immunosuppressive response after rituximab is usually delayed and occurs after 6 to 12 weeks (3). The patient had two ECT per week, with bilateral electrode stimulation and high energy. The seizures were effective (mean 32 s). It would have been preferable to intensify the rhythm of the ECT sessions but this was impossible due to the pandemic situation.

The ECT treatment has been complicated to initiate due to two factors: first, the parents were reluctant toward this treatment and secondly, the anesthesiologists were requisitioned for the Covid-19 patients. Moreover, after the beginning of the ECT, we had to suspend the ECT treatment because of cardiologic side effects (rhythm disturbances).

## Conclusion

Cerebral ^18^F-fluorodeoxyglucose PET imaging could be considered a relevant biomarker in the assessment of possible/probable seronegative autoimmune encephalitis associated with psychiatric manifestations that is an infrequent but complex clinical presentation in child and adolescent psychiatry, as it may be the only abnormal paraclinical exam. This noninvasive imaging test could help guide the diagnosis and early treatment of AIE, significantly impacting the prognosis of this serious illness.

## Data Availability Statement

The raw data supporting the conclusions of this article will be made available by the authors, without undue reservation.

## Ethics Statement

Ethical review and approval was not required for the study on human participants in accordance with the local legislation and institutional requirements. Written informed consent to participate in this study was provided by the participants' legal guardian/next of kin. Written informed consent was obtained from the patient and his parents for publication of the case report and the attached figures, PET images and videos.

## Disclosure

During the last 2 years, DC reported past consultation for or the receipt of honoraria from Otsuka, Lundbeck, and Jansen.

## Author Contributions

MG, MR, and AC: wrote the first draft. AK: supervised the TEP logistic and produced Figure 1. AK, MS, and MB: evaluation of TEP and recruitment of TEP controls. AA, AO, and MR: were in charge of the psychiatric treatment. DP and YA: gave neurologic and internal medicine expertise and treatment respectively. DC and AC: supervised the whole case report and revised the MS. All authors contributed to the article and approved the submitted version.

## Conflict of Interest

The authors declare that the research was conducted in the absence of any commercial or financial relationships that could be construed as a potential conflict of interest.
